# Role of PPAR*α* and Its Agonist in Renal Diseases

**DOI:** 10.1155/2010/345098

**Published:** 2010-11-08

**Authors:** Ching-Feng Cheng, Hsi-Hsien Chen, Heng Lin

**Affiliations:** ^1^Department of Medical Research, Tzu Chi General Hospital and Department of Pediatrics, Tzu Chi University, Hualien 970, Taiwan; ^2^Institute of Biomedical Sciences, Academia Sinica, Taipei 115, Taiwan; ^3^Graduate Institute of Clinical Medicine, Taipei Medical University and Department of Internal Medicine, Taipei Medical University Hospital, Taipei 110, Taiwan; ^4^Graduate Institute of Pharmacology and Toxicology, Tzu Chi University, 701 Chung Yang Road, Section 3, Hualien 970, Taiwan

## Abstract

Peroxisome proliferator-activated receptor (PPAR)-*α*, a member of a large nuclear receptor superfamily, plays a major role in the regulation of lipid metabolism. Recently, PPAR*α* activation has been shown to confer additional benefits on endothelial function, kidney function, and anti-inflammation, suggesting that PPAR*α* agonists may be good candidates for treating acute renal failure. In clinical application, PPAR-*α* activators, such as hypolipidemic drugs in fibric acid class, were proven to have therapeutic effects on metabolic syndrome and cardiovascular disease. This paper focuses on signaling pathways, ligand selectivity, and physio-pathological roles of PPAR*α* in kidney diseases and the therapeutic utility of PPAR*α* modulators in the treatment of diabetes and inflammation-induced nephropathy. Implication of new and more potent PPAR-*α* activators could provide important insights into the overall benefits of activating PPAR-*α* clinically for the treatment of dyslipidemia and the prevention of diabetic or inflammation-induced nephropathy in the future.

## 1. Peroxisome Proliferator-Activated Receptors

Peroxisome proliferator-activated receptors (PPARs) are nuclear hormone receptors, that is, ligand-dependent intracellular proteins that stimulate transcription of specific genes by binding to specific DNA sequences. When activated by appropriate ligand binding, their transcription factors affect development and metabolism. There are three PPAR subtypes, products of the distinct genes commonly designated as PPAR*α*, PPAR*γ*, and PPAR*β*/*δ*, or merely *δ* [1]. The PPARs usually heterodimerize with another nuclear receptor, the 9-cis-retinoic acid receptor (RXR), forming a complex that interacts with specific DNA-response elements within the promoter regions of the target genes. Ligand binding can activate this heterodimer complex which recruits transcription coactivators and regulates the transcription of genes involved in the regulation of lipid and carbohydrate metabolism [1]. Like several other nuclear hormone receptors, it heterodimerizes with RXR to form a transcriptionally competent complex [2].

## 2. Tissue Expression of PPARs and Their Role in Renal Injury

PPAR*α*, PPAR*β*/*δ*, and PPAR*γ* are differentially expressed in various tissues [3–5]. In general, PPAR*α* is highly expressed in tissues that possess high mitochondrial and *β*-oxidation activity, including the liver, renal cortex, intestinal mucosa, and heart, with lower expression in several other tissues. PPAR*γ* is highly enriched in adipose tissue, while lower expression levels are reported in the urinary bladder, intestine, kidney, spleen, adrenal, heart, liver, lung, brain, and vasculature. Unlike PPAR*α* and PPAR*γ*, low-level expressions of PPAR*β*/*δ* is ubiquitously found in almost every tissue examined. In the kidney, PPAR*α* is abundantly expressed in the proximal tubules and the medullary thick ascending limbs with much lower expression in the glomerular mesangial cells [5, 6]. PPAR*γ* is primarily expressed in the distal medullary collecting ducts, with lesser expression in the glomeruli and renal microvasculature [7]. In the kidney, PPAR*β*/*δ* is diffusely expressed in the renal cortex and medulla, including medullary interstitial and stromal cells [5]. This differential tissue distribution of the three PPAR isoforms may be related to their distinct roles in these tissues, including the kidney. Because the target genes of PPAR*α*, -*β*/*δ*, and -*γ* in these tissues are mainly involved in adipogenesis, lipid metabolism, insulin sensitivity, glucose homeostasis, and cell growth and differentiation, PPARs could be the target candidates that modulate body metabolisms.

Prior studies in animal models had described the beneficial roles for PPARs in reducing renal injury and dysfunction. For instances, PPAR*β*/*δ* pretreatment could protect wild-type mice from renal I/R injury, with a reduction in medullary necrosis and inflammation [8]. PPAR*γ* agonists rosiglitazone and pioglitazone had shown protective effects against renal ischemia/reperfusion (I/R), diabetic nephropathy, and various kidney injury [9, 10]. Although a role for PPAR*α* in reducing renal injury and PPAR*α* ligands could attenuate cisplatin-induced acute renal failure (ARF) was reported in animal models [11, 12], its exact mechanisms are still inconclusive. Therefore, this paper will focus on the role of PPAR*α* and its agonist in renal diseases.

## 3. PPAR*α* Ligands and Their Clinical Implications

Fibric acid derivatives or fibrates are PPAR*α* ligands. Fibrates have been used in clinical practice for more than four decades to decrease triglyceride levels. Fibrates can also increase HDL cholesterol levels, with a limited but significant additional effect on decreasing low-density lipoprotein (LDL) cholesterol levels. In addition to its major effects on lipid profiles, mounting evidence shows that beneficial effects of fibrates may be due to their anti-inflammatory and antiatherosclerotic properties [13, 14]. The PPAR agonists can be synthetic molecules, such as fibrates used to treat hypertriglyceridemia or thiazolidinediones to treat insulin resistance, or natural ligands, such as fatty acids (FAs) and their derivatives (eicosanoids). Although fibrates are most efficient in patients with high TG and low HDL, marginal effects in the treatment of dyslipidemia were found in the recent ACCORD (Action to Control Cardiovascular Risk in Diabetes) trials to patients with type-2 diabetes [15]. Nevertheless, recent ACCORD studies demonstrated that fibrate therapy with intensive glycemia control could reduce renal microalbuminuria significantly [16]. Although microalbuminuria may rather be a marker for cardiovascular disease [17], its applications as a reversible marker of kidney and vascular damage were recently reported [18, 19].

## 4. PPAR*α* and Diabetic Nephropathy

Although the abundance of PPAR*α* in the kidney is well established, its role in renal physiology and diabetic nephropathy is just emerging. PPAR*α* was implicated in the regulation of kidney metabolism and to maintain a sustained balance between energy production and expenditure [20], given its high level expression in the renal proximal tubules [5, 21, 22]. Clofibrate activates PPAR*α* and induces expression of *β*-oxidation enzymes, long-chain and medium-chain acyl-CoA dehydrogenase, and acyl-CoA oxidase in the renal cortex [23]. It is suggested that renal PPAR*α* might play a major role in triggering fatty acid utilization and adaptive response to dietary lipids. This idea is further supported by a recent study in which the beneficial effects of fasting-induced upregulation of pyruvate dehydrogenase kinases were blunted in PPAR*α*-deficient mice, indicating that loss of PPAR*α* can lead to abnormal renal regulation during starvation [24]. Although PPAR*α* induction is beneficial in fasting and hyperlipidemia, effects of PPAR*α* in diabetic nephropathy remain unclear. However, clinical evidence suggests a beneficial effect of fibrate treatment in patients with type-2 diabetes [25, 26], and data from the recent FIELD (Fenofibrate Intervention for Event Lowering in Diabetes) study also indicate promising effects with fenofibrate in preventing progression of diabetes-related microvascular complications [27]. In db/db type-2 diabetic mice, treatment with fenofibrate markedly lowers urinary albumin excretion and improves glomerular mesangial expansion [28, 29]. Therefore, both clinical observations and rodent experiments suggest that PPAR*α* activation may play a beneficial role in diabetes induced nephropathy. 

## 5. PPAR*α* and Kidney Mesangial Cells

Clofibrate has been shown to inhibit oxidative stress-induced TGF-*β* expression in glomerular mesangial cells [30]. Expression of PPAR*α* in glomerular mesangial cells has also been reported [31]; thus it is likely that PPAR*α* activation in mesangial cells could block TGF-*β* signaling pathway and thereby attenuating glomerular matrix proliferation. Consistent with this suggestion, a recent study demonstrated that fenofibrate downregulates TGF-*β*1 and TGF-*β* signaling receptor II expression and decreases collagen IV deposition in the diabetic glomeruli [32]. Conversely, starved PPAR*α* null mice would show increased albuminuria with albumin accumulation in the proximal tubules further confirming the beneficial role of PPAR-*α* [33]. Therefore, it is likely that PPAR*α* activation may facilitate albumin reabsorption and degradation in the nephron segment [34, 35]. Taken together, fenofibrate treatment activated PPAR*α* may reduce TGF-*β*-induced proliferation in mesangial cells, thus ameliorate kidney injury.

## 6. Involvement of PPAR*α* in Inflammation

PPAR*α* plays a critical role as a primary sensor and regulator of lipid metabolism, and this role has increasingly been recognized to be important in inflammation-induced disorders including hypertension, metabolic disorders, cardiovascular disease, atherosclerosis, and inflammation-induced acute renal failure [36]. Fenofibrates, ligands for PPAR*α*, are used clinically to treat patients with type-2 diabetes or coronary disease [37]. Fibrates can exert anti-inflammatory effects, by decreasing plasma levels of cytokines IL-6, TNF*α*, and IFN*γ* in patients with atherosclerosis [38] or level of CRP in patients with cardiovascular diseases [39]. In human endothelial cells, PPAR*α* activators interfere with processes involved in leukocyte recruitment and cell adhesion by inhibiting the expression of VCAM-1. Since PPAR*α* agonists (fenofibric acid and eicosapentaenoic acid) enhance e-NOS expression and NO release, this suggests a vaso-protective effect. In other studies, synthetic PPAR*α* activators (fenofibric acid and WY14643) diminish thrombin-induced and oxidized LDL-induced expression of endothelin-1 [38]. PPAR*α* activators can also modify inflammatory vascular smooth muscle cells (VSMC) activation by inhibiting IL-1-induced production of IL-6 and prostaglandins and by reducing the expression of cyclooxygenase-2 (COX-2). In addition, PPAR*α* agonists reduce tissue factor and MMP expression in monocytes and macrophages. Moreover, PPAR*α* activation, in the presence of TNF*α* and IFN*γ*, may promote macrophage apoptosis. Finally, activators of PPAR*α* limit the production of proatherogenic Th1 cytokines such as IFN*γ*, TNF*α*, and IL-2 [38]. PPAR*α* activators also inhibit the inflammatory response in hepatocytes by decreasing IL-1-induced CRP and IL-6-induced fibrinogen *α*, -*β*, and serum amyloid A expression [39]. PPAR*α* thus acts as an antiatherogenic factor by modulating local and systemic inflammatory responses.

## 7. Involvement of PPAR*α* in Ischemia-Reperfusion-Induced Kidney Injury

Although the causes of ARF are often multifactorial, they can be generally classified into three categories depending on the causes: (1) prerenal ARF, in which the kidney fails to receive an adequate blood supply, for example, due to a fall in systemic blood pressure subsequent to hemorrhage [40]; (2) intrinsic ARF, in which the failure originates within the kidney, for example, due to drug-induced nephrotoxicity like traditional cisplatin or gentamicin-induced nephrotoxicity; and (3) postrenal ARF, caused by impairment of urine flow from the kidney, for example, due to ureteral obstruction or bladder/prostate cancer. Increasing evidence supports a role for PPAR*α* in the development of ARF. Several studies have demonstrated a reduction in PPAR*α* expression, transcriptional activity, and inhibition of peroxisomal and mitochondrial fatty acid oxidation (FAO) enzymes in rodent renal tissue undergoing cisplatin- and I/R-induced ARF [41]. Activation of PPAR*α* with ligands such as fibrate or WY14643 reduces cisplatin and I/R-induced acute kidney injury [42]. Importantly, these effects of fibrate and WY14643 are not observed in PPAR*α*-null mice. These mice subjected to I/R injury by arterial ligation show enhanced cortical necrosis and impaired renal function [22]. However, such renal I/R injury could be rescued via induction of PPAR*α* with recovery of normal kidney structure and function [22]. Recent investigations using kidney androgen-induced protein 2 (KAP2) promoter with tissue-restricted expression model further corroborate the essential role of PPAR*α* in renal protection [43]. As KAP2 is exclusively expressed in the proximal tubules under the control of androgens, their studies delineated that the androgen-induced proximal tubules PPAR*α* transgenic mice could afford protection against cisplatin- and I/R-induced inhibition of FAO and protected kidney function and morphology from these insults, in comparison with their effects on wild-type mice. In addition, the organ and tissue (proximal tubule-) restricted expression model in their studies further ruling out the potential PPAR*α*-independent, renoprotective actions as well as excluding the potential PPAR*α*-mediated, extrarenal effects in renal protection afforded by PPAR*α* activators in the PPAR*α*-null mouse [43]. We also demonstrated that prostacyclin may act as an inducer, which can enhance PPAR*α* translocation into the nucleus and bind to inflammatory transcriptional factor NF*κ*B thus inhibiting TNF*α*-induced apoptosis in renal epithelial cells. In addition, wild-type mice pretreated with a PPAR*α* activator, docosahexaenoic acid (DHA), could significantly reduce I/R-induced renal dysfunction (lowered serum creatinine and urea nitrogen levels), apoptotic responses (decreased apoptotic cell number and caspase-3 and -8 activation), and NF-*κ*B activation [33]. Altogether, these studies strongly endorse a critical role of PPAR*α* in the preservation of renal morphology and function during cisplatin- or I/R-induced acute renal damage.

## 8. Regulation of PPAR*α*

**Figure 1:**
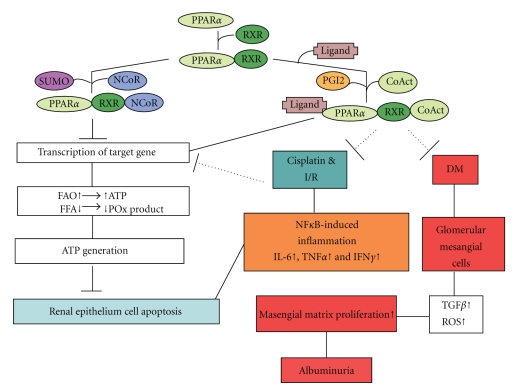
Schematic diagram presenting the signaling pathways of PPAR*α* involved in the mechanisms of ischemic/reperfusion-, drugs-, or diabetic-induced renal damage. PPAR*α* forms heterodimer with RXR. In the absence of ligands, the dimer may recruit a corepressor, inhibiting PPAR*α*-mediated transcription of target genes. The presence of an agonist, or an activator such as PGI2, triggers the recruitment of a coactivator complex which induces transcriptional activity of PPAR*α* onto its target genes. This leads to an increase in fatty acid catabolism and adenosine triphosphate (ATP) production, also to decrease the levels of cytotoxic fatty acid peroxidation (PO_x_) products, and, consequently, to promote cell viability and inhibit renal epithelium cell death. In addition, PPAR*α* complex can attenuate NF*κ*B-induced inflammatory factors (IL-6, INF*γ*, or TNF*α*) induced by ischemic/reperfusion injury (I/R) or drugs. Furthermore, PPAR*α* complex can inhibit masengial matrix proliferation induced by TGF*β* or reactive oxidative stress (ROS) which then resulted in albuminuria. After SUMOylation of PPAR*α*, SUMOylated PPAR*α* resulted in downregulation of its transcriptional activity by promoting its interaction with the corepressor NCoR, which will compromise cell viability and activate cell death processes. CoAct, coactivator; DM, diabetes mellitus; FAO, fatty acid oxidation; FFA, free fatty acid; IFN*γ*, interferon *γ*; IL-6, interleukine-6; I/R, ischemia/reperfusion; NCoR, nuclear corepressor; NF-*κ*B, nuclear factor-*κ*B; PGI2, prostacyclin; POx, peroxidation; PPAR*α*, peroxisome proliferator-activated receptor-*α*; RXR, retinoid X receptor; TGF*β*, tumor growth factor *β*; TNF*α*, tumor necrosis factor *α*.

Ligands binding to PPAR*α* unmask an interaction area (of PPAR*α*) for coactivators such as cAMP response element-binding protein (CREB-) binding protein (CBP)/p300. The latter possesses histone acetyl transferase (HAT) activity resulting in chromatin decondensation and PPAR*α* heterodimerization with RXR. The binding of this heterodimer to PPRE on PPAR*α* promoter then regulates target genes expression. In addition, PPAR*α*(s) are substrates for several kinases activated by a variety of endogenous or exogenous signals. These kinase include: extracellular receptor kinase-mitogen-activated protein kinase (ERK-MAPK), JNK and p38 MAPK, Protein kinase A, Protein kinase C (PKC), 5′-AMP-activated protein kinase (AMPK), and glycogen synthase kinase 3 (GSK3). Recently, SUMOylation of PPAR*α* has reported that SUMOylated hPPAR*α* on lysine 185 resulted in down-regulation of its transcriptional activity by promoting its interaction with the corepressor NCoR [44]. Therefore, it is interesting to investigate whether PPAR*α* modification, including phosporylation, SUMOylation, and ubiquitination, is involved in inflammation-induced renal failure. Recently, we also demonstrated that adiponectin exerts protective effect against renal ischemic-reperfusion injury via prostacyclin- PPAR*α*-heme oxygenase-1 signaling pathway (unpublished data). A schematic diagram presenting the regulation of PPAR*α* in renal disease is depicted in Figure 1.

## 9. Conclusion and Perspectives

PPAR*α*, in the last few years, has emerged as the key regulator of lipid homeostasis in *in vitro* experiments and clinical medicine. In addition, PPAR*α* negatively regulates inflammation-mediated phenomenon like atherosclerosis and ARF. PPAR*α* ligand and fibrates are pharmacologic agents with pleiotropic effects. Fibrates have beneficial effects in alleviating cardiovascular abnormalities, ARF-, diabetic- or drug-induced nephropathy, in both animal models and clinical trials [45, 46]. Although the effects of PPAR*α* have not been fully investigated, they are shown to be protective in chronic kidney diseases.
